# Phylogenetic analysis of glycoprotein B gene sequences of bovine herpesvirus 1 isolates from India reveals the predominance of subtype 1.1

**DOI:** 10.14202/vetworld.2016.1364-1369

**Published:** 2016-12-05

**Authors:** S. S. Patil, A. Prajapati, D. Hemadri, K. P. Suresh, G. S. Desai, G. B. Manjunatha Reddy, B. M. Chandranaik, S. Ranganatha, H. Rahman

**Affiliations:** 1 ICAR-National Institute of Veterinary Epidemiology and Disease Informatics, Bengaluru - 560 064, Karnataka, India; 2 Institute of Animal Health and Veterinary Biologicals, Bengaluru - 560 024, Karnataka, India; 3 Division of Animal Sciences, Indian Council of Agriculture Research, Krishi Bhawan, New Delhi - 110 001, India

**Keywords:** bovine herpesvirus 1-1, glycoprotein B, India, infectious bovine rhinotracheitis, phylogenetic analysis, sequencing, subtype 1.1

## Abstract

**Aim:**

This study was conducted for the isolation and molecular characterization of bovine herpesvirus 1 (BoHV-1) isolated from the nasal and vaginal swabs collected from naturally infected cattle showing clinical symptoms of the respiratory disease.

**Materials and Methods:**

Isolation of BoHV-1 virus performed on clinical samples collected from 65 cattle from five states of India. The BoHV-1 isolates were further confirmed by polymerase chain reaction (PCR) using primers specific for glycoprotein B (gB) genomic region. PCR amplification was performed using previously published gB gene-specific primer pairs. gB PCR amplicons obtained from all isolates were sequenced, and phylogenetic analysis was performed using software.

**Results:**

A total of 12 samples were found positive in cell culture isolation. 11 isolates showed the visible cytopathic effect on Madin-Darby bovine kidney after 72 h. Partial sequence analysis of gB gene of all isolates revealed 99.0-100% homology between them. All isolates showed 99.2-99.8% homology with Cooper stain.

**Conclusion:**

BoHV-1.1 is the predominant circulating subtype of BoHV in India, and all isolates have homology with Cooper stain.

## Introduction

Infectious bovine rhinotracheitis (IBR) is a contagious disease of cattle and buffaloes caused by bovine herpesvirus Type 1 (BoHV-1) which is belonging to genus *Varicellovirus* in the subfamily Alphaherpesvirinae under the family *Herpesviridae* [1,2]. The virus is responsible for severe economic losses to the dairy industry worldwide due to abortions leading to increased calving interval, reduced milk yield, weight loss, and restrictions on international livestock trade [2]. Clinically disease manifests as conjunctivitis, red nose, abortions and reduction in milk yield [3]. BoHV-1 genome encloses 10 genes encoding glycoproteins, among them six are in the upper long (UL) segment whereas the remaining four ones are in the upper short segment [4]. Based on the genomic analysis and restriction endonuclease analysis, BoHV-1 can be divided into three subtypes: BoHV-1.1 (respiratory infections), BoHV-1.2a (genital infections), and BoHV-1.2b (encephalitis) [5].

From the first report of IBR in India [6], several authors have reported varying seroprevalence rates ranging from 40.00% to 60.46% in Indian cattle population [7-13]. All subtypes are antigenically similar and only established methods of genetic characterization are able to identify the prevalence of BoHV-1 types and subtypes [14]. Many attempts of isolation of BoHV-1 have been made by Indian researchers [6,14-16]. Some researchers used partial sequencing of the UL27 gene to evaluate the genetic variation of BoHV-1 isolates [16]. UL27 gene codes for glycoprotein B (gB), one of the immunonodominant antigens present in the viral envelope, is involved in virus attachment, entry, direct cell-to-cell spread, and fusion [3]. It induces strong neutralizing antibody response [17,18] and is recognized by CD4+ helper T lymphocytes [19] making it a potential candidate for subunit vaccine and recombinant protein-based enzyme-linked immunosorbent assay. gB gene has sufficient variability to generate high-resolution phylogenetic trees to divide the virus into different groups and provide more information about the transmission and distribution of this virus [16].

The aim of this study was to generate baseline information about Indian BoHV-1 subtypes based on gB gene sequences and also to increase our understanding about the genetic relatedness of Indian BoHV-1 with other bovine alphaherpesviruses. Such studies are needed to determine circulating strain of virus which can help in the development of vaccine to provide proper protection to susceptible animals.

## Materials and Methods

### Ethical approval

The study was duly approved by the Institutional Animal Ethics Committee of National Institute of Veterinary Epidemiology and Disease Informatics, Bengaluru.

### Sample collection

Nasal and vaginal swabs samples were collected from suspected cattle (sign of fever, tearing, serous ocular or nasal secretion, and history of recent abortion) from the farms located in Karnataka, Tamil Nadu, Gujarat, Orissa and Uttar Pradesh (Table-1). The nasal and vaginal swabs were dipped in Eagle’s minimum essential medium (EMEM) (Gibco, Germany) containing antibiotics 100 IU/mL penicillin and 100 µg/mL streptomycin, vortexed and centrifuged at 1000 g for 10 min. Madin-Darby bovine kidney cells (MDBK) monolayers used for virus isolation were obtained from Virology Laboratory of the Institute

**Table-1:** Details of the samples used in the study.

States	Animal numbers	Type of sample collected
Karnataka	26	Nasal and vaginal swab
Tamil Nadu	14	Nasal and vaginal swab
Gujarat	10	Nasal and vaginal swab
Orissa	8	Nasal and vaginal swab
Uttar Pradesh	7	Nasal and vaginal swab
Total	65*	

* 65 each nasal and vaginal samples=130 samples

### Virus isolation

Virus isolation was carried out as per the procedure described in OIE manual (2010). Briefly, 1 ml of media soaked with clinical swabs that were centrifuged and syringe filtered (Millipore) were inoculated onto a monolayer of MDBK cells and incubated for 1 h at 37°C for adsorption. Then, inoculum was decanted and the MDBK cells monolayer washed with maintenance medium (EMEM containing 50 mg/ml of gentamicin). After washing, 5 ml of maintenance medium was added and the infected cells were incubated at 37°C and observed for 4-5 days for any changes in the cells. Three such consecutive passages were carried out till the sample is discarded. The infected cells were harvested when the cytopathic effects (CPE) like rounding of cells, clustering of cells appearing as bunch of grapes are observed. The infected cells were freeze thawed 3 times, centrifuged at 1200 ×*g* for 10 min to remove cellular debris. The BoHV-1 isolates were further confirmed by polymerase chain reaction (PCR) using primers specific for gB genomic region. Total DNA was extracted from the supernatant of infected cells using a QIAamp DNA mini kit (Qiagen, Germany), and the DNA was subjected to PCR using previously published gB gene-specific primer pairs (IBR443F: 5’-tcgaargccgagtacctgcg-3’ nt position 56051-56070 of AJ004801 and IBR 443R: 5’-ccagtcccaggcraccgtcac-3’ nt position 56494-56474 of AJ004801) [20]. PCR was carried out in a total of 25 µl reaction volume containing: 2.5 µl of ×10 PCR buffer, 2.5 µl (2.5 mM) of MgCl_2_, 1.0 µl (10 mM) of deoxynucleotide triphosphate mix, 1.0 µl (10 U/µl) of Taq DNA polymerase enzyme, 1.0 µl (25 pmol) each of above mentioned forward and reverse primers. The final volume was adjusted with nuclease free water. The PCR amplification was carried out with the initial denaturation at 95°C for 5 min and 35 cycles each of denaturation at 94°C for 50 s, annealing at 58°C for 50 s, and extension at 72°C for 50 s. One cycle of the final extension was carried at 72°C for 5 min. The PCR amplified products were visualized on 1.0% agarose gel. The purity of the extracted DNA was checked by spectrophotometer. The PCR products were gel purified (QIA Quick Gel Purification Kit, Qiagen, Germany) and subjected to cycle sequencing at both strands from a commercial service provider.

### Sequence analysis

The obtained sequences were and aligned using “Sequencher 5.1” software (https://genecodes.com) followed by BLAST analysis in GenBank database for comparing with other BoHV 1 sequences. The sequences of representative BoHV isolates and related herpesvirus were retrieved from GenBank (http://www.ncbi.nlm.nih.gov/Blast) and were included in the phylogenetic analysis. Sequence analysis was conducted using the Editseq and Megalign programs of the Lasergene 6 package (DNAstar Inc., Madison, USA). The nucleotide sequence alignment was used to create a neighbor joining phylogenetic tree. Percent frequencies of the groupings were determined after 1000 bootstrap evaluations, and the tree was viewed using the Tree View program [21]. The nucleotide alignment produced by the ClustalX program was used to generate the sequence distances using the Megalign program of the DNAstar package.

## Results

Out of 130 samples processed, 12 virus isolates were recovered in cell culture. 11 samples showed characteristic CPE in the first passage after 72-96 h of inoculation while 1 sample gave CPE in the second passage. The percentage of recovery of virus isolates is more in nasal swabs (10/65) than vaginal swabs (02/65) and it does not recover from same animals. All isolates (nasal and vaginal) were confirmed to be a 1.1 subtype based on nucleotide sequence analysis of gB genomic region. Partial sequence analysis of gB gene of all isolates revealed 99.0-100% homology between them. All sequences from India showed 99.2-99.8% homology to the reference Cooper strain sequence. Percentage similarity of all isolates varies from 99.0% to 99.2% to BoHV-1.2, 95.0-95.5% to BoHV-5 and 95.5-95.8% to bubaline herpesvirus 1 (BuHV-1). The phylogenetic analysis showed all isolates form different subgroup with Australian BoHV-5 reference strain N569 and located in a distinct branch. All the isolates showed 39.7-40.1% and 41.9-42.3% of divergence with Porcine herpesvirus 2 (PHV-2) and canine herpesvirus 2 (CHV-2) reference strain, respectively. Similar to the nucleotide sequences, deduced amino acid sequences revealed a high degree of identity in all the BoHV-1 isolates used in the study.

## Discussion

IBR causes respiratory, genital and nervous diseases in cattle with high morbidity and low mortality rates [3]. BoHV-1 induces carrier state in the infected animals, due to which the presence of antibodies in an animal does not indicate the true picture of active infection [4]. Infected and carrier animals (particularly under stress conditions) tend to shed the virus intermittently in nasal, ocular, vaginal secretions, and in semen. Considering this major drawback in serum based tests [4], detection of virus becomes mandatory to designate an animal as positive for BoHV-1.

Growth of virus in cell culture confirmed by bunch of grape-like clustering, rounding of cell and presence of giant cells which was in correlation with previous BoHV-1 isolation studies [22] and the number of days to produce visible CPE differed for most samples. All the samples were collected from the suspected animals showing clinical signs of IBR. Shedding of virus in secretion is more during the clinical phase of disease which increases the sensitivity of virus isolation and PCR [23,24].

PCR is an invaluable tool for fast and sensitive detection of BoHV-1 sequences in biological and clinical specimens [25]. PCR amplification of target gene with gB specific primers showed the 443 bp single product in agarose gel electrophoresis.

Earlier, different subtypes of BoHV-1 were identified based on restriction enzyme analysis of purified virus DNA [14] but it requires purified and large quantity of DNA. With the advent of PCR and nucleic acid sequencing, many researchers used this technique for determination of subtypes of BoHV-1 based on sequencing of gC, gD, and gB gene [15,24,26,27]. The gB of BoHV-1 is one the most important glycoproteins because of it functional properties (virus entry, viral gene expression, replication, and phylogenetic relationships) [16]. Many researchers used amplification and sequencing of gB gene of BoHV1 for diagnosis and understanding the molecular epidemiology and disease distribution of disease in India and worldwide [16,24,28,29].

**Figure-1:**
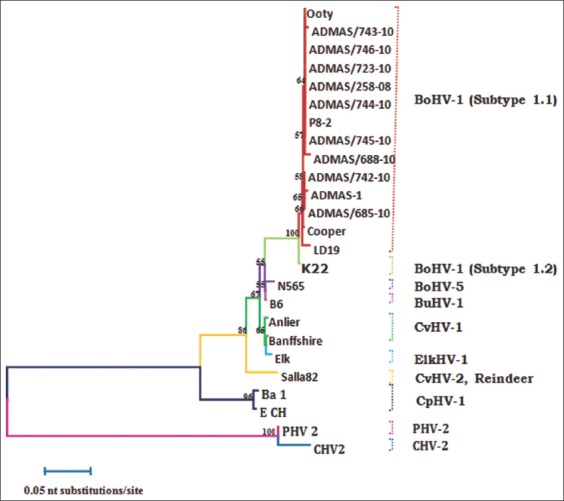
Neighbor joining tree showing grouping of Indian bovine herpes virus 1 isolates with other herpes viruses. The tree was displayed using the Tree View program. The numbers at each branch indicate the % frequency of this evaluation after 1000 bootstrap evaluations. All sequences, excluding isolated, were obtained from the NCBI PubMed website.

All isolates were confirmed to be a 1.1 subtype is also supported by results of Ravishankar *et al*. [15], Surendra *et al*. [16], Sreenivasa *et al*. [30] who demonstrated that BoHV 1.1 is the common subtype in India. Subtypes 1.1 and 1.2 of BoHV-1 usually infect respiratory and genital tract of cattle, but many reports found that each subtype can be better adapted to either respiratory or genital tract [26,31]. Although a product length of 443 nucleotides (nts) was generated by PCR, only 400 nts corresponding to the position from 56,086 to 56,485 of Cooper strain (AJ004801) was aligned and used for nt sequence analysis due to non availability of that stretch of sequence from many other types/subtypes. Aligned nt sequences revealed high degrees of identity in all alpha herpesviruses compared in the study. Partial nucleotide sequence analysis of gB was performed to understand the genetic similarity of isolated IBR virus with other virus strains circulating in India and other parts of the world. Partial sequence analysis of gB gene of all isolates revealed 99.0-100% homology between them. Similarity percentage of all isolates varies from 99.0% to 99.2% (BoHV-1.2), 95.0-95.5% (BoHV-5), and 95.5-95.8% (BuHV-1). These findings are concurrent with recent finding of Surendra *et al*. [16] who reported gB based sequencing of Indian isolates showed high similarity among them (99.11-100%) and 98.45-99.34% with BoHV-1.2. All the isolates showed 39.7-40.1% and 41.9-42.3% of divergence with PHV-2 and CHV-2 reference strain respectively. Similar to the nucleotide sequences, deduced amino acid sequences revealed a high degree of identity in all the BoHV-1 isolates used in the study (Figure-1).

**Figure-2:**
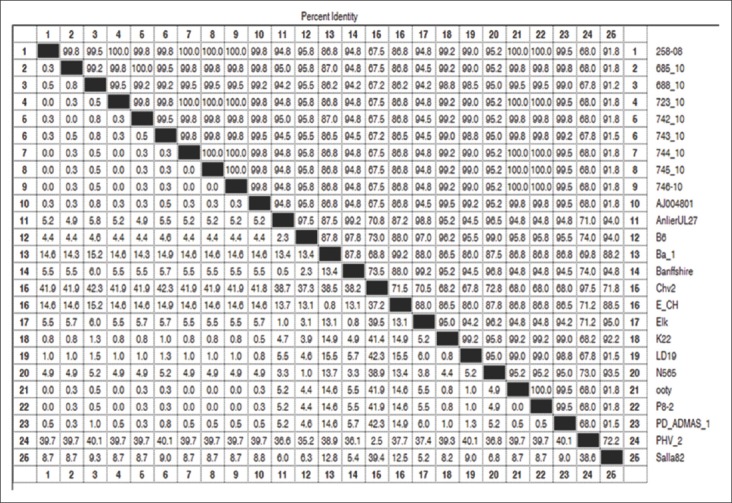
Glycoprotein B gene sequence pair distance map of bovine herpes virus 1 *isolates* calculated by DNA Star software.

Partial nucleotide sequences of gB gene showed similar topology of clustering for BoHV-1, BoHV-2, BoHV-5, BuHV and other related herpesvirus as described by earlier reports [32,33]. Phylogenetic analysis of nucleotides of gB region clustered our all isolate with BoHV subtype 1.1 with other respiratory isolates and separated from genital strains (BoHV-1.2) and other related alpha herpesvirus (caprine herpesvirus 1, cervid herpesviruses 1, BuHV-1, CHV-1, and PHV-1). None of the Indian isolates of this study closely related to BoHV-5. So far no report of isolation of BoHV-5 is available in India. This result also supports previous finding that BoHV-5 is not circulating in Indian cattle [16]. Further, analysis of many isolates from various parts of India is required to establish the presence or absence of BoHV-5. BoHV-5 is very closely related to BoHV-1 sharing a high degree of genomic and antigenic homology [34]. Nucleotides differences present along the genome of both the virus may be useful for specific differentiation at type and subtype level [35,36]. Although Saha *et al*. [33] reported the presence of BoHV-1.2 subtype in the nasal swab of Indian cattle, none of the isolates (nasal and genital swab) of this study could be classified as BoHV-1.2. It might be due to conserved nature of gB that is unable to differentiate between BoHV-1.1 and BoHV1.2 (Figure-2) [24].

## Conclusion

It has been now established that IBR is endemic in India and produce huge economic losses to dairy industry. In India, inspite of huge potential, IBR vaccine is not being developed and not in use. To achieve IBR free status, better vaccines are needed with companion tests. Most of phylogenetic analysis studies of BoHV-1 restricted to characterization of gC gene. This is one of kind of approach to characterize gB gene to understand molecular epidemiology of IBR using 12 virus isolates. Further studies with such molecular characterization of more viruses collected from different geographical location of India will help to have a better understanding of the epidemiology and development of new vaccine and diagnostics.

## Authors’ Contributions

SSP conceptualized the aim of the study, designed, planned, and supervised the experiments and corrected the manuscript. GSD, GBMR, BMC and SR performed the isolation and molecular biology work. AP and KPS drafted the manuscript. DH and HR provided conceptual support, and critically reviewed the manuscript. All authors read and approved the final manuscript.
